# Radiofrequency ablation for selective fetal reduction in complicated Monochorionic twins; comparing the outcomes according to the indications

**DOI:** 10.1186/s12884-021-03656-1

**Published:** 2021-03-06

**Authors:** Fatemeh Rahimi-Sharbaf, Marjan Ghaemi, Ahmed A. Nassr, Alireza A. Shamshirsaz, Mahboobeh Shirazi

**Affiliations:** 1 Yas Hospital, Tehran University of Medical Sciences, Tehran, Iran; 2 Valie-Asr Reproductive Health Research Center, (VRHRC), Tehran University of Medical Sciences, Tehran, Iran; 3 Baylor College of Medicine, Houston, Texas USA; 4 Maternal, Fetal and Neonatal Research Center, Tehran University Of Medical Sciences, Tehran, Iran

**Keywords:** Monochorionic Diamniotic pregnancy, Twin-twin transfusion syndrome, Twin reversed arterial perfusion sequence, Selective fetal growth restriction, Fetal reduction

## Abstract

**Background:**

To evaluate the perinatal outcomes in women with complicated monochorionic diamniotic twins who underwent selective reduction using radiofrequency ablation (RFA).

**Methods:**

This retrospective study included patients with complicated monochorionic diamniotic twins between 16 to 28 weeks who underwent selective reduction using RFA.

**Results:**

During the study period, 143 women with complicated monochorionic twins underwent RFA including 52 with selective fetal growth restriction (sFGR), 48 with twin to twin transfusion syndrome (TTTS), 33 with major fetal anomalies in one of the twins, and 10 with reversed arterial perfusion sequence (TRAP). The overall survival was 71.3% (102/143). The procedures were technically successful in achieving selective termination in all cases. The mean ± SD of gestational age at the time of the procedure was 21.0 ± 2.3 weeks. The mean ± SD of gestational age at delivery was 34.6 ± 3.3 weeks. The mean ± SD of overall procedure-to-delivery time was 12 ± 1.7 weeks. The pregnancy success rates among sFGR, TRAP, TTTS and anomaly groups were 82.7, 80, 73 and 60.7% respectively. There were no maternal complications.

**Conclusion:**

Radiofrequency ablation for fetal reduction in complicated monochorionic twin pregnancies appears to be a reasonable option. The pregnancy success rate following RFA selective reduction was highest among sFGR and TRAP groups and lowest in the anomaly group.

## Introduction

Monochorionic twins are associated with specific complications due to placental sharing including higher risk of fetal growth restriction, twin-twin transfusion syndrome (TTTS), early preterm delivery and perinatal mortality. Structural anomalies are also encountered more commonly in monochorionic compared to dichorionic twins [1].

These conditions may cause death of the most affected twin and severe morbidity or mortality of the co-twin. It has been reported that death or major insult to the brain and other organs of the co-twin can be encountered after single fetal death in approximately 15 and 25% of cases respectively due to sudden exsanguinations through vascular anastomoses towards the dead twin [2].

TTTS management includes expectant management, amnioreduction, laser ablation, septostomy and selective fetal termination using techniques such as umbilical cord occlusion. In some cases, the treatment aim is to enable one twin to survive as the chances for both surviving are extremely poor. Some parents may choose to terminate the pregnancy because of the high risk of perinatal morbidity and mortality in both twins [3].

Selective feticide is one of the treatment options to minimize the potential neurological injuries and to improve the chances of survival of the normal co-twin in complicated monochorionic pregnancies [4, 5].

Currently, fetoscopic laser coagulation of the vascular anastomoses in the placenta is the selected management for twin-twin transfusion syndrome. However, radiofrequency ablation could be an alternative option in cases in which laser is technically not feasible or in the cases with severe cerebral injury, congenital abnormalities or severe fetal growth restriction in donor or recipient twin [6].

In the current study, we describe the perinatal outcomes of 143 consecutive cases of complicated monochorionic twin pregnancies that underwent selective reduction with radiofrequency ablation (RFA) at our institution according to the indications for the intervention.

## Materials and methods

### Study criteria

This retrospective study included all pregnant women with complicated monochorionic diamniotic twins who underwent selective reduction using RFA between 16 to 28 weeks’ gestation.

### Ethics

This study was approved by the institutional Ethical Committee (code IR.TUMS.VCR.REC.1396.4772) and was carried out between June 2016 and September 2018 at Yas hospital, a tertiary referral center for fetal medicine, affiliated to Tehran University of Medical sciences, Tehran, Iran. Informed consent was taken from all participants. This trial was conducted according to the principles of the Helsinki Declaration.

### Indications

Indications for RFA were complicated monochorionic diamniotic twins including TTTS (*n* = 48) (stage II, *n* = 15; stage III, *n* = 29; stage IV, n = 4), sFGR (*n* = 52) (type II, n = 15; type III, *n* = 37), TRAP (*n* = 10) and major structural anomalies in one of the twins (*n* = 33).

All patients underwent aneuploidy screening and if they were at high risk, amniocenteses were performed. Detailed anatomic survey and fetal echocardiography were also performed in all cases. Amnioreduction was done for associated polyhydramnios if needed. Selective FGR was defined as an estimated fetal weight < 10 th percentile in one twin and/or an inter-twin estimated fetal weight discordance of > 20% [7]. According to the Delphi procedure in MC twins, at least two out of four contributory parameters (EFW of one twin <10th centile, abdominal circumference of one twin <10th centile, EFW discordance of ≥25%, and umbilical artery pulsatility index of the smaller twin >95th centile) were agreed and in DC twins, at least two out of three contributory parameters (EFW of one twin <10th centile, EFW discordance of ≥25%, and umbilical artery pulsatility index of the smaller twin >95th centile) were accepted [8]. Totally, we choose ≥20% as cut off for discordance.

TTTS was staged according to the staging system proposed by Quintero et al. [9]. We mostly choose the smaller twin except for hydrops cases. As in stage II TTTS we have we encountered the lowest mortality rate, but because the parents emphasized the complete health for the fetus, they preferred to feticide so that the complications would be minimal for the live fetus.

Thirty minutes before the intervention, amoxicillin 1000 mg for infection prophylaxis, and indomethacin rectal suppository 50 mg for tocolysis were administered and repeated after six hours.

### Technique

All RFA procedures were performed under local anesthesia supplemented by intramuscular sedation (50 mg pethidine and 25 mg promethazine).

Under continuous ultrasound guidance, the radiofrequency needle 17-gauge, (RF medical MYGEN made in South Korea) was inserted percutaneously and through the uterus into the abdominal umbilical cord insertion site of the abnormal fetus.

We tried to cross umbilical vein (Targeted vessel for ablation). Then the radiofrequency energy was applied until a temperature of 100 C^O^ was delivered for 1 to 2 min. An area representing the zone of thermal injury was then easily seen on ultrasound at the end of the procedure. Usually, 2 to 3 cycles were required before complete cessation of blood flow was achieved.

All patients were examined one week after the procedure in our hospital or at the local hospital. Follow up was continued until delivery, by contacting the patients or their physicians.

Statistical analysis was performed using software SPSS v22.0 software. Categorical data were expressed as number/proportion and analyzed by X2-test or Fisher’s exact test.

## Results

Over the study period, 143 RFA procedures were performed. The mean gestational age at the time of the procedure was 21.0 ± 2.3 weeks. The overall procedure-to-delivery time was 12 ± 1.7 weeks. The procedures were technically successful (cessation of blood flow in abnormal fetus) in all the cases.

There were no maternal complications such as chorioamnionitis, sepsis, and hemorrhage, admission to the intensive care unit or maternal death.

Overall, 28.6% (41/143) of cases were complicated by fetal demise of the co-twin. Out of the cases, fetal demise was diagnosed the next day in 29.2% (12/41), within one week in 12.1% (5/41) and after one week (up to a maximum of 84 days) of the RFA procedure in 58.5% (24/41) of cases.

Mean gestational age at the time of delivery was 34.6 ± 3.3 days. Overall, the live birth rate was 71.3% (102/143).

In preterm live birth group, delivery was due premature preterm rupture of the membrane (PPROM) in 42.8% (24/56) and preterm labor in 53.5% (30/56) of cases. Two cases were delivered at 29- and 34.7-weeks’ gestation due to placenta abruption and both survived the neonatal period. The outcomes of the RFA according to indications are summarized in Table 1. The maternal age, parity, risk factors for prematurity, gestational age at procedure and length of procedure were not significantly different between the groups.

**Table 1:** The obstetric outcomes of RFA procedures according to the indications

	Total N (%)	TTTS N (%)	sFGR N (%)	Anomaly N (%)	TRAP N (%)	P
**Numbers**	**143**	**48**	**52**	**33**	**10**	
**PPROM**	24 (16.7)	10 (20.8)	11 (21.2)	2 (6.0)	1 (10.0)	0.233
**Preterm Labor**	30 (20.9)	10 (20.8)	13 (25.0)	5 (15.1)	2 (20.0)	0.731
**Mean GA at delivery (weeks)**	34.6	33.6	34.6	36.1	34.5	0.012
**GA at birth 24–31 6/7**	39 (27.2)	15 (31.2)	17 (32.6)	5 (15.1)	2 (20.0)	0.872
**32–36 6/7 weeks**	37 (25.8)	15 (31.2)	13 (25.0)	6 (18.1)	3 (30.0)	0.450
**> 37 weeks**	46 (32.1)	12 (25.0)	19 (36.5)	12 (36.3)	3 (30.0)	0.488
**Mean GA at procedure (weeks)**	21.0	21.0	21.6	19.8	21.5	0.027
**Perinatal mortality**	41 (28.6)	14 (29.1)	13 (25.0)	11 (33.3)	3 (30)	0.576
**Co-twin death ≤ 1 week after RFA**	15 (10.4)	6 (12.5)	3 (5.7)	5 (15.1)	1 (10.0)	0.351
**Co-twin death > 1 week after RFA**	28 (19.5)	9 (18.7)	10 (19.2)	6 (18.1)	3 (30.0)	0.955
**PPROM < 34 weeks**	12 (8.3)	10 (20.8)	1 (1.9)	1 (3.0)	0 (0)	< 0.001
**GA at RFA ≤ 20 weeks**	46 (32.1)	13 (27.0)	13 (25.0)	18 (54.5)	2 (20.0)	0.216
**GA at RFA > 20 weeks**	97 (67.9)	35 (63.0)	39 (75.0)	15 (45.5)	8 (80.0)	0.942

TTTS, twin to twin transfusion syndrome; sFGR, selective fetal growth restriction; TRAP, twin reversed arterial perfusion sequence

## Discussion

Our study suggested that RFA is an acceptable method for fetal reduction in complicated monochorionic diamniotic twins with a success rate of more than 70%. The pregnancy success rate following RFA selective reduction was highest among sFGR and TRAP groups and lowest in the anomaly group. Radiofrequency was the only method that used in Iran between 2016 and 2018. Recently, the bipolar coagulation and interstitial laser have been introduced and applied experimentally in clinical trials.

We report live birth rate of approximately 71% that is almost the same as previously reported [10–12]. In our cohort, the obstetric outcomes were better in TTTS compared to sFGR cases. This could be related to the hemodynamic alterations that affect both twins in cases of TTTS, in addition to the risk preterm delivery associated with severe polyhydramnios [13]. In TRAP cases, Zhang et al. reported a survival rate of 70% for the pump twin in cases treated by RFA [14] which is approximately the same as noticed in our cohort.

PPROM and preterm labor were the most common complications that lead to unfavorable consequences in the neonates [15]. Similarly in our study, 75% of live-births born preterm due to the previously mentioned reasons. It is believed that laser may have more PPROM as unwanted outcome, because radiofrequency ablation is acted only in the internal body of the fetus; otherwise, the laser fetoscopic coagulation is applied on placental vascularization that is may be close to or in touch with the fetus membrane, and make it more prone to rupture [6].

In our study, the lowest pregnancy success rate was noted in the anomaly group, which contradicted the observation reported by Sun et al. [16]. The lower perinatal survival rate in this group may be related to the technical challenges resulting from normal volume of amniotic fluid.

Thermal injuries to fetus have been reported after RFA [17]. In our cohort, we had one fetus with leg’s burn that was born at 31 weeks’ gestation. This neonate underwent plastic surgery and expired at 3 days of life. We had no cases of thermal injury to uterus.

In our study, we encountered late fetus demise despite the successful RFA procedure. The etiology is unknown and we did not find a specific reason for it. Perhaps one reason is for the late visit, despite the success of the process, the fetus demises due to the pre-existing complications.

Technical options for selective feticide include bipolar cord occlusion, fetoscopic laser cord coagulation or RFA. Fetoscopic laser photocoagulation is the optimal management for TTTS in second trimester of gestation. And the outcomes is improving in twin survival. But the risk of neurologic sequel is remained about 11–14% [18].

On the other hand RFA has potential benefits over other modalities because the diameter of the umbilical cord is relatively small, making the procedure minimally invasive and applicable in early gestations as well. Consequently, RFA is characterized by high survival rates for the co-twin and low rates of preterm premature rupture of the membranes [15, 19, 20].

The strengths of our study are the large sample size and standardized technique used in the management of all cases. Main limitation of our study was the absence of long-term outcomes.

Since the laser ablation is a relatively new and acceptable method in the management of the complicated twins, one of the limitations of our study is that the laser was recently launched in our center at the time of the study, and therefore due to the possible technical problems, this method was not applied in our patients.

Due to the fact that most patients who referred to our center were from the other cities from all over the country, it was not possible for them to come back for weekly visit. They followed the perinatal visit by their own obstetrician and periodic biometry. We had called them by phone to follow them up due to the high cost of the autopsy and their distance from our center, this process was not done for the demised fetuses.

## Conclusion

In conclusion, radiofrequency ablation for fetal reduction in complicated monochorionic twin pregnancies appears to be a reasonable option. The pregnancy success rate following RFA selective reduction was highest among sFGR and TRAP groups and lowest in the anomaly group.

The awareness regarding the frequency and focus of follow-up ultrasound examinations after RFA. In the absence of any maternal or fetal complications at 1 and 2 weeks after the procedure, the patient is often discharged to the referring center, where the obstetrical care is left to the discretion of the referring gynecologist. In most cases, further follow-up ultrasound appointments will be in accordance with the care for uncomplicated singleton pregnancies.

The lower perinatal survival rate among the anomaly group may be related to the technical challenges resulting from normal volume of amniotic fluid. Alternative techniques such as bipolar coagulation may be more appropriate in these cases.

## Data Availability

The datasets used during the current study are available from the corresponding author on reasonable request. They are divided in two group. The data of the patients that declared in the article and are available with more detail by corresponding author and can be sent by her.

## Abbreviations

RFA: Radiofrequency ablation
sFGR: Selective fetal growth restriction
TTTS: Twin to twin transfusion syndrome
TRAP: Reversed arterial perfusion sequence
SD: Standard deviation
PPROM: Premature preterm rupture of the membrane
